# The importance of viral blips and duration of therapy initiated in primary infection in maintaining viral control after stopping cART

**DOI:** 10.7448/IAS.17.4.19820

**Published:** 2014-11-02

**Authors:** Sarah Fidler, Ashley Olson, Julie Fox, Andrew Phillips, Charles Morrison, John Thornhill, Heiner Bucher, Roberto Muga, Kholoud Porter

**Affiliations:** 1 Department of Genitourinary Medicine and Infectious Diseases, Imperial College, London, UK; 2 Institute of Clinical Trials & Methodology, University College London, London, UK; 3 School of Life and Medical Sciences, University College London, London, UK; 4 Behavioral and Biomedical Research Division, Family Health International, Durham, NC, USA; 5 University Hospital Basel, Basel Institute for Clinical Epidemiology, Basel, Switzerland; 6 Department of Internal Medicine, Hospital Universitari Germans Trias i Pujol, Badalona, Spain

## Abstract

**Introduction:**

After achieving undetectable HIV-RNA on cART, on cessation, HIV-RNA rebounds to pre-treatment values for the majority due to the presence of an inaccessible viral reservoir. There is some evidence that cART during primary HIV infection (PHI) limits the reservoir size, optimizing the chance of maintaining viral control off cART. Data are required to predict possible viral controllers for treatment interruption following cART. This analysis aims to investigate the effect of cART duration and the rate of viral blips while on cART initiated in PHI, and other factors on maintaining viral control for those stopping cART.

**Material and Methods:**

Using CASCADE data on HIV seroconverters, we characterized virologic blip (viral suppression on cART followed by a single HIV-RNA above a blip threshold and a subsequent measure below the threshold without cART change) rates for those starting cART within six months of seroconversion (SC). Using Cox models, we examined the effect of the following factors on time to virologic rebound (HIV-RNA>1000) after cART stop: cART duration, severity/rate of blips on cART, time from SC to cART start, cART class, SC year, SC age, CD4 at cART start/stop, sex and HIV risk group.

**Results:**

The 660 individuals initiating cART in PHI were mostly male (91%), seroconverting between 1995 and 2012, with a median (IQR) age of 34 (29, 41) years mostly infected through sex between men (73%). Median cART duration was 14.8 (7.0, 31.7) months initiated at a median 1.9 (0.5, 3.9) months post SC. 13 (11, 16), 9 (7, 11), 6 (5, 9) and 7 (6, 10)% of individuals experienced blips >50, 100, 200 and 400 copies/mL, respectively. Of those who experienced blips, most (77–90%, depending on blip threshold) experienced just one. Among 250 individuals with undetectable HIV-RNA at cART stop, median time to rebound was 1.6 (0.30, 5.8) months. Time on cART was the only factor independently associated with control after stopping, HR for rebound=0.91 (0.86, 0.98) per extra six months spent on cART, HR for ever blipping >400 copies/mL while on cART=0.88 (0.40, 1.93).

**Conclusions:**

Blips occur in about 10% of individuals who initiate cART in PHI, most of who experience only one blip, but was not predictive of subsequent virologic rebound. Increasing time spent on cART initiated in PHI could increase time of virological suppression after cART stop.

